# Work-related injuries among Syrian refugee child workers in the Bekaa Valley of Lebanon: A gender-sensitive analysis

**DOI:** 10.1371/journal.pone.0257330

**Published:** 2021-09-20

**Authors:** Rima R. Habib, Diana Mikati, Josleen Al-Barathie, Elio Abi Younes, Mohammed Jawad, Khalil El Asmar, Micheline Ziadee

**Affiliations:** 1 Faculty of Health Sciences, American University of Beirut, Beirut, Lebanon; 2 Faculty of Medicine, American University of Beirut, Beirut, Lebanon; 3 Public Health Policy Evaluation Unit, Imperial College London, London, United Kingdom; University of Tripoli, LIBYA

## Abstract

**Background:**

Syrian refugees in Lebanon have endured increasing hardships since the onset of the Syrian war in 2011, with many resorting to child labor. Working refugee children endure socioeconomic deprivation and harsh working conditions. This study explores the relationship between working conditions and the reporting of injuries among male and female Syrian refugee children in Lebanon and the related gender differences.

**Methods and findings:**

A cross-sectional survey of Syrian refugee children working in the Bekaa Valley of Lebanon was conducted in 2017. Face-to-face interviews with children (8 to 18 years) collected sociodemographic information and testimonies of their work experiences. Logistic regression tested the association between reporting of injuries and risk factors including school enrolment, field of work, means of transportation to work, age started working, number of working hours, multiple jobs, work pressure and hazards, and abuse. Analyses were stratified by gender.

Of the 4090 surveyed working children, the majority reported working in agriculture (75.8%). Around a third (31.4%) reported being injured at work with a higher proportion in males. The most common reported injuries were cuts and wounds (44.9%), with males showing a higher proportion for all types of injuries compared to females. Nearly one fifth of reported injuries (19.8%) required medical attention in a hospital, with males reporting higher proportions than females for most types of injuries. The study findings revealed the association of multiple risk factors with an increased odds of reporting an injury, which included working in more than one job (AOR, 1.71; CI, 1.20–2.43; p = 0.003), working under pressure (AOR, 1.64; CI, 1.36–1.97; *p*<0.001), the use of sharp or heavy objects (AOR, 1.88; CI, 1.58–2.24; *p*<0.001), and experiencing physical abuse at work (AOR, 2.46; CI, 1.97–3.08; *p*<0.001). The odds of reporting an injury increased with every additional hour of work per day (AOR 1.08; CI, 1.02–1.14; *p* = 0.006). Most of these findings persisted in the male and female stratified models, with few exceptions. Males who went to work in a pickup truck had significantly lower odds of being injured than those who walked (AOR, 0.65; CI, 0.51–0.83; p = 0.001); this finding did not reach significance for females. Having longer work hours per day was significantly linked to higher odds of injury for females (AOR, 1.07; CI, 1.02–1.12; p = 0.008); but not for males. The main limitations of this study were its cross-sectional design and the use of self-reported variables.

**Conclusions:**

This study is the first to obtain direct testimony on work-related injuries and working conditions, exploring gender differences, among Syrian refugee children in Lebanon. Results demonstrated the association between the occurrence of injury and multiple risk factors highlighting their strenuous working conditions, with some differences detected between males and females. Many injuries can be prevented through direct safety interventions and proper implementation of child labor policies. Multidimensional interventions are essential to address the complex evolving challenges facing refugees.

## Introduction

Since the beginning of the war in Syria in March 2011, more than a million displaced Syrians have taken refuge in Lebanon [1], particularly in the Bekaa Valley bordering Syria, making it a main settling ground for displaced Syrians. The Bekaa, which constitutes 42% of Lebanon’s cultivated land, has long been a destination for Syrian migrant workers who crossed the border seasonally seeking agricultural work for a temporary stay in Lebanon [2]. Following the Syrian war, the cross-border labor migration ceased, and Syrians became permanent refugees in Lebanon [2]. Today, many Syrian refugee families living in the Bekaa send their children to work in agriculture as well as various other sectors [3]. Many live in extreme poverty in informal tented settlements, lacking basic needs and proper housing, rendering them one of the most vulnerable populations in the country [3].

Regulations imposed by the Lebanese government to control the residence and work of Syrian refugees in Lebanon have also contributed to the exacerbation of child labor. As of May 2015, Syrian nationals could no longer register as refugees with the United Nations High Commissioner for Refugees (UNHCR) [4]. The complicated registration process to obtain a residence or work permit has made it more difficult for refugees to live and work legally in the country [5]. This multitude of restrictions has forced refugees to either move back home or to succumb to the taxing processes to secure their stay in Lebanon [5]. To overcome the financial burdens and precarious living conditions, many refugee families have resorted to sending their children to work [6–8].

While child labor is considered a violation of children’s rights, for displaced families, it is rather a form of self-sufficiency [6,9]. Some of the major determinants of child labor include poverty, displacement, large family size, and illiterate heads of households [10,11]. Families in underprivileged populations rely on their children as a key source of financial security [11–18]. These children are often withheld from school and put to work due to poverty and the perception that education will not directly improve the quality of their life; on the other hand, working from a young age would help them gain experience and skills, which in turn would provide them with a better future [6]. Accordingly, children take on precarious jobs with difficult working conditions in sectors such as agriculture and construction. Employers exploit the inexperienced unprotected children whom they can easily control and employ for lower wages [9–11,14]. As a result, children become trapped in a vicious cycle of poverty; their work compromises their education and that puts them at a greater risk for poverty.

Child labor is a worldwide phenomenon but is remarkably prevalent in low-income countries [18,19]. Many initiatives have emerged to eradicate child labor and alleviate its negative outcomes. The International Labor Organization (ILO), which has been committed to the abolition of child labor for the past 100 years, issued a number of conventions in pursuit of the abolition of child labor [20,21]. Additionally, one of the major targets of the United Nations’ (UN) Sustainable Development Goals (SDGs) is the termination of child labor [12,22]. Leading the task of eliminating child labor globally, the ILO designated 2021 as the year to achieve this goal, by aiming to “take immediate and effective measures to eradicate forced labor, end modern slavery and human trafficking and secure the prohibition and elimination of the worst forms of child labor, including recruitment and use of child soldiers, and by 2025 end child labor in all its forms” [23].

In Lebanon, the Ministry of Labor has attempted to curtail child labor and declared its commitment to the ILO’s recommendations by issuing a National Action Plan (NAP) to eliminate the worst forms of child labor in 2013 [24]. After the influx of refugees, the Lebanese government adjusted the existing action plan in 2017 by including Syrian refugee child laborers, with the aim to eradicate the worst form of child labor by 2020 [25]. However, many such efforts have been insufficient, and recent reports show that child labor remains a problem in Lebanon [17,26].

At work, children are exposed to hazards subjecting them to immediate threats, long-term health consequences, and psychological harm. Being physiologically fragile and unsuited to endure working with machinery or equipment designed for adults, children are at increased risk for physical harm which can be prevented [9,11,14,15,18,19,27]. Physical and ergonomic hazards can include falling objects, working in extreme temperatures, dangerous machinery, load lifting, awkward postures, poor ventilation, unhygienic conditions, and exposure to toxins [10–12,16,18,28,29]. Physical abuse and harassment are also common causes of bodily harm reported among child workers [10,17,18]. In addition, negative health outcomes such as injuries, eyestrain, headache, gastrointestinal problems, musculoskeletal symptoms, and skin diseases, as well as premature death have been reported among working children [10,11,14–16,27,29].

Literature on work-related injuries among children has explored associations with various risk factors including age, gender, working hours, and type of work [12,22]. A number of studies revealed that male child workers are at a higher risk of injuries than their female counterparts [11,12,16,30]. Other studies also show that injuries are more prevalent among those who work for long hours [9,11,12,19,31,32], work in agriculture, retail trades, or construction [9,12,17,31–33], and those who accept poor working conditions for slightly higher wages [27]. Moreover, injuries sustained by working children are not limited to those that happen in the workplace, but also include injuries that occur during the commute to work [34]. While the literature has amassed evidence on the hardships of child workers, recent studies highlight the need for additional research on the adverse outcomes of child labor in low and middle income countries [22], particularly in the context of humanitarian settings such as that of Syrian refugees [17]. Child labor continues to be a primary concern in Syrian refugee communities in Lebanon [26]. Recent reports have documented that Syrian refugee children endure socioeconomic deprivation and extremely harsh working conditions [17,26] with physically demanding work and hazardous exposures [17,29].

Using a gender lens, our study aims to analyze the work-related injuries and associated risk factors among Syrian refugee child workers in the Bekaa Valley, where the majority of Syrians who sought refuge in Lebanon reside. We adopted a gender-sensitive approach [35–37] to reveal differences between males and females in work experiences, exposures, and associated risk of injuries.

## Methods

### Design, setting, and sampling

This study is based on a cross-sectional household survey of Syrian refugee children actively working in the Bekaa Valley of Lebanon in 2017 [17]. The study was developed using a detailed protocol to guide the design [37,38], and was approved by the Institutional Review Board (IRB) at the American University of Beirut (IRB Protocol Number: FHS. RH1.08). It follows the Strengthening the Reporting of Observational Studies in Epidemiology (STROBE) guidelines (See S1 STROBE checklist). The study methodology is also described in recently published articles [3,17,38,39].

The sampling frame was based on the Interagency Mapping Platform (IAMP), a database used for the coordination of humanitarian activities including information on Syrian refugees residing in informal tented settlements (ITSs) in Lebanon, with geographical locations, settlement size, and number of tents and refugees per settlement [40]. We randomly sampled 153 ITSs out of 3748 listed in the Bekaa. We were able to identify households with working children between 8 and 18 years in the selected ITSs, with the help of a local community gatekeeper, also known as the “Shaweesh”, involved in the daily affairs of Syrian refugees living in ITSs and in connecting them with employers to find work [41]. Data was collected during a three-month period between 14 August 2017 and 27 November 2017, with the help of 27 Arabic-speaking Lebanese and Syrian fieldworkers recruited through a local non-governmental organization working with refugee children and trained for the data collection. A pilot was carried out in an ITS that was not selected in the study sample to test the questionnaire and identify needed adjustments. The fieldworkers contacted 1907 households, of which 1902 households agreed to participate. We surveyed 4090 male and female child workers between 8 and 18 years and collected detailed data on their demographics and work experience. The high response rate was due to the coordination with local community gatekeepers during the preparation for the fieldwork and implementing the fieldwork in collaboration with non-governmental organizations well known by the study population. In addition, each household was revisited three times to find the working children, which ensured maximum participation.

### Questionnaire and measures

A questionnaire was administered face-to-face to the working children by the trained fieldworkers in colloquial Arabic. The fieldworkers obtained oral informed consent from the female homemaker in each household and assent from the working children, explaining the purpose of the study and emphasizing the confidentiality of the collected data. They also explained that participation was voluntary and refusal to participate will not lead to any retaliatory actions. Supervisors, recruited to oversee the adequate implementation of the fieldwork activities, observed the administration of the questionnaires and the consent process. The data were collected electronically on a tablet using KoBo Toolbox [42]. Working children provided direct responses to the questionnaire (See S1 Text), which contained questions on demographic information, including age, gender, attending school, age started working, and the number of years since the child started working, as well as work characteristics including field of work, tasks performed in agricultural work, average work hours per day, working in more than one job, wages based on piece-rate pay, working under pressure to finish the job on time, taking breaks during the work day, exposure to physical abuse, and the use of sharp or heavy objects, in addition to method of transportation to work (walking, cycling, pick-up truck). In the survey, children answered questions on whether they had ever experienced an injury at work (Yes/No), which was the primary outcome measure for this study. They also provided information on the type of injury they suffered and whether the injury required medical attention (hospitalization) as a proxy indication of the severity of the injury [31]. Based on the Occupational Injury and Illness Classification System (OIICS) Manual (Version 2.01) of U.S. Bureau of Labor Statistics [43], the types of injuries included those caused by (1) falls/slips/trips, (2) electrocution/burns, (3) falling objects; in addition to (4) cuts and wounds, (5) fractures/sprains, (6) eye injuries, and (7) insect/animal bites.

### Statistical analysis

Descriptive statistical analysis was carried out by reporting means and standard deviations (SDs) for continuous variables, and frequencies and percentages for categorical variables. Chi squared and independent samples t-tests were performed to test for significant differences between males and females. Logistic regression was used to model the association between the main outcome variable (ever had an injury at work) and the risk factors, including, the number of years since the child started working, gender, attending school, average work hours per day, working in more than one job, piece-rate pay, working under pressure to finish job on time, taking breaks during work day, use of sharp/heavy objects at work, physically abused at work, and transportation to work, adjusting for age and for the effect of clustering at the household level. We also stratified our model by gender (See S1 Table for males and S2 Table for females). We conducted the analysis using Stata V.15.0 and we considered an alpha value of 0.05 to be statistically significant.

## Results

Table 1 presents the sociodemographic and work characteristics of the 4090 children (8–18 years) in the study sample. The average age was 13.2 years (SD = 2.7) and about half (51.5%) were males. The mean age of starting work was 11.2 years (SD = 2.6), similar in both genders. The children had been working for 2 years on average (SD = 1.5), and they worked for around 6.7 hours per day (SD = 3.0). Only 18.2% of children were attending school at the time with more males than females (19.7% of males compared to 16.5% of females). Around 4.9% of children had more than one job, which was more common for males than females (7.1% and 2.6% respectively).

**Table 1:** Sociodemographic and work characteristics of Syrian refugee working children (8–18 years) in the Bekaa Valley, Lebanon, 2017 (N = 4090).

	Total		Male		Female	
**Total, N (%)**	4090 (100%)		2107 (51.5%)		1983 (48.5%)	
**Characteristics**	**Mean**	**SD**	**Mean**	**SD**	**Mean**	**SD**
**Age, years**	13.2	2.7	13.1	2.7	13.4	2.7
**Age started working, years**	11.2	2.6	11.1	2.6	11.4	2.6
**Number of years since the child began to work**	2.0	1.5	2.0	1.4	2.0	1.5
**Average work hours/day**	6.7	3	6.9	3.3	6.5	2.6
	**N**	**%**	**N**	**%**	**N**	**%**
**Age group, years**						
8–12	1482	36.2	813	38.6	669	33.7
13–15	1466	35.8	737	35.0	729	36.8
16–18	1142	27.9	557	26.4	585	29.5
**Attending school**	744	18.2	416	19.7	328	16.5
**Working in more than one job**	202	4.9	150	7.1	52	2.6

Abbreviations: SD, standard deviation.

Table 2 details work-related injury across age groups. Around 1283 children (31.4%) reported being injured at work with the highest proportion (38.3%) for children in the age group 13–15 years. Males reported more injuries than females (58.7% in males compared to 41.3% in females).

**Table 2:** Syrian refugee working children (8–18 years) reporting work-related injury across age groups in the Bekaa Valley, Lebanon, 2017 (N = 4090).

	Injured Working Children		Male		Female		*p-*value**
Total injured, N (%)	1283* (31.4%)		753 (58.7%)		530 (41.3%)		<0.001
	N	%	N	%	N	%	
**Age (years)**							
8–12	400	31.2	238	31.6	162	30.6	<0.001
13–15	491	38.3	281	37.3	210	39.6	
16–18	392	30.6	234	31.1	158	29.8	

*The number of children who reported ever being injured (N = 1283).

**p-values for the differences in the distribution between males and females.

In Table 3, the distribution of Syrian refugee working children (8–18 years) and those reporting work-related injury across field of work, showed that the majority of the children worked in agriculture (75.8%), followed by waste picking (4.2%), vending and delivery (4.1%), among other occupations. Most females (85.8%) worked in agriculture followed by craft and related trade work (5.4%). Among males, a majority but not as many (66.3%) worked in agriculture, followed by construction (7.0%), which was exclusive to males along with mechanics (3.7%). The highest proportions of injury across field of work were reported in miscellaneous jobs (64.5%), mechanics (52.3%) and factory work (50.6%). Males showed significantly higher proportions of injury compared to females for all fields of work except street services (32.3% in males, and 44.4% in females).

**Table 3:** Distribution of Syrian refugee working children (8–18 years) and those reporting injuries across field of work in the Bekaa Valley, Lebanon, 2017 (N = 4090).

	Working Children						Injured Working Children						
	Total		Male		Female		Total		Male		Female		p-value*
	N	%	N	%	N	%	N	%	N	%	N	%	
	4090	100	2107	51.5	1983	48.5	1283	31.4	753	58.7	530	41.3	
**Field of work^a^**													
Agriculture	3098	75.8	1396	66.3	1702	85.8	881	28.4	432	31.0	449	26.4	<0.001
Construction	147	3.6	147	7.0	0	0	64	43.5	64	43.5	0	0.00	
Craft and related trade work	167	4.1	60	2.9	107	5.4	54	32.3	25	41.7	29	27.1	
Vending and delivery	168	4.1	123	5.8	45	2.3	54	32.1	45	36.6	9	20.0	
Waste picking	173	4.2	121	5.7	52	2.6	54	31.2	43	35.5	11	21.2	
Factory work	87	2.1	51	2.4	36	1.8	44	50.6	29	56.9	15	41.7	
Mechanics	78	1.9	78	3.7	0	0	41	52.6	41	52.6	0	0.0	
Street services	98	2.4	62	2.9	36	1.8	36	36.7	20	32.3	16	44.4	
Car wash	100	2.4	97	4.6	3	0.2	35	35.0	35	36.1	0	0.0	
Miscellaneous jobs^b^	31	0.8	28	1.3	3	0.2	20	64.5	19	67.9	1	33.3	

^a^Total adds up to more than 100% as more than one option is possible.

^b^ Denotes various jobs based on availability.

* p-values for the differences in the distribution between males and females.

Table 4 shows the types of work-related injuries (N = 2128) and hospitalization reported by the injured working children (N = 1283) by gender. The most common injuries reported by the children were cuts and wounds (44.9%), followed by injuries caused by falls/trips/slips (14.7%), eye injuries (9.7%), and fractures/sprains (9.2%). The reporting of injuries was higher in males than in females for all types of injury. Nearly one fifth of reported injuries (19.8%) required medical attention in a hospital, with the highest proportion for fractures/sprains (38.8%) and injuries caused by falling objects (34.9%). Males reported higher proportions of injuries that required medical attention than females for all types of injuries except those caused by electrocution/burns and insect bites.

**Table 4:** Type of work-related injury and hospitalization among male and female Syrian refugee working children (8–18 years) in the Bekaa Valley, Lebanon, 2017 (N = 2128*).

	Total number of injuries		Hospitalized injuries		Number of injuries among males		Hospitalized injuries among males		Number of injuries among females		Hospitalized injuries among females	
**Total, N (%)**	2128		421	19.8	1335	62.7	288	21.6	793	37.3	133	16.8
**Mean (SD) number of types of injuries per child**	1.66 (1.1)		-	-	1.78 (1.2)		-	-	1.50 (0.9)		-	-
**Type of injury**	**N**	**%**	**N**	**%**	**N**	**%**	**N**	**%**	**N**	**%**	**N**	**%**
Cuts and wounds	955	44.9	185	19.4	602	63.0	123	20.4	353	37.0	62	17.6
Injuries caused by Falls/Slips/Trips	313	14.7	42	13.4	199	63.6	31	15.6	114	36.4	11	9.7
Eye injuries	206	9.7	30	14.6	116	56.3	24	20.7	90	43.7	6	6.7
Fractures/sprains	196	9.2	76	38.8	132	67.4	53	40.2	64	32.7	23	35.9
Injuries caused by electrocution/burns	184	8.7	16	8.7	119	64.7	9	7.6	65	35.3	7	10.8
Insect/Animal bite	139	6.5	25	18.0	73	52.5	11	15.1	66	47.5	14	21.2
Injuries caused by falling objects	129	6.1	45	34.9	89	69.0	35	39.3	40	31.0	10	25.0
Injury caused by vehicle accident	6	0.3	2	33.3	5	83.3	2	40.0	1	16.7	0	0.0

*The total number of injuries reported by children (N = 2128). A child can report one or multiple injuries.

Table 5 shows the prevalence of injury across work tasks performed in agriculture by gender. The majority of children were engaged in harvesting followed by loading/carrying, weeding, and cultivating. The highest proportion of injuries was reported among children engaged in planting (47.9%). Both males and females showed similar prevalence of injuries in harvesting (24.7% in males, 23.5% in females), and loading/carrying (36.1% in males, 38.4% in females). On the other hand, male children had higher injury proportions when engaged in weeding (47.4% in males, 31.4% in females), cultivating (27.0% in males, 16.1% in females), and planting (55.6% in males, 44.8% in females). Whereas females reported higher proportions of injury for food sorting activities than males (35.5% in females compared to 30.2% in males). Injuries incurred by children performing loading/carrying tasks showed higher proportions of hospitalization compared to the other agricultural tasks such as livestock keeping and planting. The differences in injuries requiring medical attention between males and females were not statistically significant; only cultivating tasks showed a borderline significance with a higher proportion for males than females (25.8% in males, 5.6% in females).

**Table 5:** Prevalence of injury across tasks in agriculture for male and female Syrian refugee working children in the Bekaa Valley, Lebanon, 2017 (N = 3098*).

	Total					Male					Female					*p-*value**
	Children Performing Task	Injured		Hospitalized Injuries		Children Performing Task	Injured		Hospitalized Injuries		Children Performing Task	Injured		Hospitalized Injuries		
Task in Agriculture	N	N	%	N	%	N	N	%	N	%	N	N	%	N	%	
Harvesting	1795	430	24.0	85	19.8	683	169	24.7	33	19.5	1112	261	23.5	52	19.9	0.539
Loading/Carrying	488	178	36.5	42	23.6	415	150	36.1	39	26.0	73	28	38.4	3	10.7	0.717
Weeding	245	83	33.9	17	20.5	38	18	47.4	6	33.3	207	65	31.4	11	16.9	0.055
Cultivating	227	49	21.6	9	18.4	115	31	27.0	8	25.8	112	18	16.1	1	5.6	0.046
Food sorting	177	60	33.9	11	18.3	53	16	30.2	4	25.0	124	44	35.5	7	15.9	0.495
Planting	94	45	47.9	8	17.8	27	15	55.6	2	13.3	67	30	44.8	6	20.0	0.343
Livestock keeping	25	10	40.0	1	10.0	23	9	39.1	1	11.1	2	1	50.0	0	0.0	0.763
Task not reported	47	26	55.3	9	34.6	42	24	57.1	8	33.3	5	2	40.0	1	50.0	0.466
**Total**	**3098**	**881**		**182**	** **	**1396**	**432**		**101**	** **	**1702**	**449**		**81**	** **	** **

* The majority of children worked in agriculture (N = 3098; 75.8%).

** *p*-value for the Chi-square test for the association between gender and injury while performing a task.

Among injured children, only 18.9% were attending school, 8.0% had more than one job, and 16.1% received piece-rate wages. While the majority (83.0%) reported taking breaks during the workday, many (67.2%) also reported working under pressure to finish the job on time, and almost half (47.9%) used sharp/heavy objects at work. Physical abuse was also prevalent in around a quarter of the injured working children (23.5%) (Table 6). Several of these risk factors were significantly associated with reporting an injury among the working children (Table 6). The number of years since the child started working showed a significant positive association with the prevalence of injury; for every additional year of work, the odds of reporting injury increased (AOR, 1.13; CI, 1.07–1.19; p<0.001). Male child workers were at significantly higher odds of reporting injury compared to females (AOR, 1.36; CI, 1.16–1.59; *p*<0.001). Children who were attending school at the time of the survey had higher odds of reporting an injury (AOR, 1.27; CI, 1.02–1.59; *p* = 0.031) than those who were not attending school. Compared to those who walked to work, children who commuted to work in a pickup truck were at lower odds of reporting an injury (AOR, 0.70; CI, 0.57–0.85; *p*<0.001). Children working in more than one job at the time of the survey were at significantly higher odds of reporting injury than those with one job only (AOR, 1.71; CI, 1.20–2.43; *p* = 0.003). In addition, there was a slight increase in the odds of reporting of injury in children who received piece-rate pay compared to those who did not; however, this result did not reach statistical significance (AOR, 1.21; CI, 0.97–1.52; *p* = 0.096). The pressure experienced at work was also associated with injuries among the working children, whereby children who reported working under pressure to finish their job on time had higher odds of injury (AOR, 1.64; CI, 1.36–1.97; *p*<0.001) than those who did not work under pressure. The odds of reporting an injury increased with every additional hour of work per day (AOR 1.08; CI, 1.02–1.14; *p* = 0.006). Taking breaks during the workday was protective against reporting an injury; however, this result was not significant (AOR, 0.94; CI, 0.76–1.17; *p* = 0.571). Another working condition that showed significant association with injury was the use of sharp or heavy objects; children who reported exposure to such materials at work were at higher odds of reporting an injury (AOR, 1.88; CI, 1.58–2.24; *p*<0.001) than those who did not. Finally, children who experienced physical abuse at work had significantly higher odds of reporting an injury (AOR, 2.46; CI, 1.97–3.08; *p*<0.001) than those who did not report abuse.

**Table 6:** Associations between sociodemographic, work characteristics and work-related injuries among Syrian refugee working children (8–18 years) in the Bekaa Valley, Lebanon, 2017 (N = 4090).

Injured (1283, 31.4%)			
		Unadjusted OR (95% CI) (*p*-value)	AOR (95% CI) (*p*-value)*
**Independent variable**			
	**Mean (SD)**		
**Number of years since child started working**	2.2 (1.5)	1.16 (1.11–1.21) (<0.001)	1.13 (1.07–1.19) (<0.001)
**Average work hours/day**	7.4 (2.8)	1.13 (1.11–1.16) (<0.001)	1.08 (1.02–1.14) (0.006)
	**% (N)**		
**Gender**			
Female	41.3 (530)	1	1
Male	58.7 (753)	1.52 (1.33–1.74) (<0.001)	1.36 (1.16–1.59) (<0.001)
**Attending school**			
No	81.1 (1041)	1	1
Yes	18.9 (242)	1.07 (0.90–1.27) (0.45)	1.27 (1.02–1.59) (0.031)
Transportation to work†			
Walking	67.7 (858)	1	1
Cycling	2.2 (28)	1.97 (1.15–3.38) (0.01)	1.59 (0.92–2.76) (0.096)
** Pickup truck**	30.1 (381)	0.85 (0.74–0.99) (0.03)	0.70 (0.57–0.85) (<0.001)
**Working in more than one job†**			
No	92.0 (1180)	1	1
Yes	8.0 (102)	2.34 (1.76–3.11) (<0.001)	1.71 (1.20–2.43) (0.003)
**Piece-rate pay**			
No	83.9 (1077)	1	1
Yes	16.1 (206)	1.13 (0.94–1.35) (0.20)	1.21 (0.97–1.52) (0.096)
**Working under pressure to finish job on time**			
No	32.8 (421)	1	1
Yes	67.2 (862)	2.02 (1.76–2.32) (<0.001)	1.64 (1.36–1.97) (<0.001)
**Taking breaks during workday**			
No	17.0 (218)	1	1
Yes	83.0 (1065)	0.86 (0.72–1.02) (0.09)	0.94 (0.76–1.17) (0.571)
**Use of sharp/heavy objects at work**			
No	52.1 (668)	1	1
Yes	47.9 (615)	2.21 (1.93–2.53) (<0.001)	1.88 (1.58–2.24) (<0.001)
**Physically abused at work**			
No	76.5 (982)	1	1
Yes	23.5 (301)	2.59 (2.17–3.09) (<0.001)	2.46 (1.97–3.08) (<0.001)

*Model clustered at the household level and adjusted for age.

†Due to respondents answering ‘I don’t know’ or respondents not answering, the total does not add up to 1283.

Abbreviations: OR: Odds ratio; AOR, adjusted odds ratio; CI, confidence interval; SD: Standard deviation.

Stratifying the logistic regression model by gender showed similar associations for injury and risk factors except for a few minor differences between males and females (S1 Table for males and S2 Table for females). For example, having more than one job showed a significant association with an increased odds of work-related injury in males (AOR, 1.62; CI, 1.07–2.46; *p =* 0.023); this association was only borderline significant in females (AOR, 1.87; CI, 0.98–3.56; *p =* 0.056). In addition, transportation to work via pickup truck showed significant association, with decreased odds of injury in males (AOR, 0.65; CI, 0.51–0.83; *p =* 0.001), but it was not significant in females (AOR, 0.79; CI, 0.56–1.10; *p =* 0.165). On the other hand, longer working hours per day was significantly associated with increased odds of injury in females (AOR, 1.07; CI, 1.02–1.12; *p* = 0.008) but only showed borderline significance in males (AOR, 1.08; CI, 0.99–1.17; *p* = 0.085).

## Discussion

To our knowledge, this is one of the largest studies globally to obtain direct testimony on the work experiences and conditions of working refugee children. The vast majority of surveyed children were not attending school at the time of the survey, with some working in more than one job. This is likely due to the deteriorating socio-economic conditions of Syrian refugee families, which compel children to set aside their education and prioritize work opportunities in support of their families [17]. This is confirmed by studies reporting lower levels of educational attainment in working children compared to non-working children, consequently jeopardizing their future prospects [10,14]. Some reports have also shown that males report higher proportions of school enrolment, reflecting the prioritization of male education over female education in impoverished communities [26]. In fact, a large proportion of children in this study started working as early as 11 years of age, with children spending an average of 6.7 hours per day at work; similar findings have been reported among populations of migrant youth workers [44]. This presents a violation of the Lebanese Labor Law and Decree 8987, which prohibits the work of children below the age of 14 and work in hazardous occupations for those below the age of 16 years; while it only permits work in such occupations for those between 16 and 18 years of age “under appropriate occupational health and safety conditions” [45,46].

Agriculture appeared as the main sector of employment for the working children. Many refugees are allowed to live in camps in exchange for their agricultural and other work services; a sort of “forced labor” deal made with the landowners and mediated by the *shaweesh* [47]. Consequently, children are compelled to work with their family members to meet this condition [18,47]. While agricultural work was predominant among both genders, a higher proportion of females worked in this field. In this context of deprivation, traditional gender norms that usually attribute labor-intensive jobs to males and homemaking roles to females can become secondary; instead, all family members are required to seek jobs in order to provide sustenance [29]. On the other hand, males were more engaged in other fields of work such as construction, waste picking, vending and delivery. Similar findings on the employment sectors of child laborers have been reported in other studies with the highest proportions in the agriculture sector; whereas construction work appeared almost exclusive to males [48].

The study findings showed injuries at work in a third of the working children, with the highest proportions of injury reported for mechanics, factory work, and construction. These findings concur with those in other studies that have demonstrated the highly hazardous conditions of these employment sectors and the increased risk of injury and adverse health outcomes [31]. The use of sharp tools and heavy objects is a common feature of work in construction, mechanics and other hazardous manual labour such as agriculture, which puts the children at risk of cuts and wounds and injuries caused by falling objects [14,32,48–52].

In line with published studies, males reported more injuries than females. The international literature show that gender differences in type and field of work play a role in exposing more male than female working children to hazardous working conditions; this results in more frequent and more serious injuries among males compared to females [14,29,31,53,54]. This was also reflected in higher proportions of injuries requiring medical attention in males compared to females. The involvement of males and females in different work sectors has been linked to differences in work-related risk of injury [34,50]. Similar to previous findings, our study showed that working in construction is predominantly a male occupation [48] and poses a high risk for injury among males; on the other hand, females report more injuries in crafts and other related trade work [34].

Within the field of agriculture, which employed the highest number of working children compared to other fields of work, proportions of work-related injuries across the tasks performed revealed some differences between males and females. Injuries appeared more prevalent among males working in weeding, cultivating, and planting than among females performing the same tasks. On the other hand, injuries were more prevalent among females working in food sorting activities than in their male counterparts. Some studies have noted differentiation in agricultural work tasks between males and females [31,55], but very few have attempted to explore gender variations in injuries within the context of these tasks [37,52,56,57]. Findings reveal that for children engaged in agricultural tasks such as cultivation, males are more likely to engage in risky behaviours and exercise less precaution when using dangerous machinery or tools than females, which increases their risk of injury [56,57]. This was reflected in our results, which showed that males reported a significantly higher proportion of injuries that required hospitalization when performing cultivation tasks. Hospitalized injuries across the other agricultural tasks were also more prevalent in males than in females; however, the results were not as significant.

Our study results demonstrate a positive association between a number of demographic and work characteristics and the reporting of injury. Children who had been working for a longer duration were at higher odds of reporting an injury at work. Previous research has shown higher proportions of injury in younger children due to lack of safety awareness, experience, and skills [14,18,50]. Children attending school were at higher odds of reporting injury than children receiving no schooling. A possible explanation is that children who have to work and attend school at the same time bare a double burden; they have to study and complete work tasks on time. Consequently, children are more likely to rush to complete tasks quickly. Performing these cognitively and physically demanding duties, children become more prone to stress, exhaustion, and burnout, as has been reported elsewhere, which in turn may increase the risk of work injuries [11,58]. Further research that explores the time distribution of injury occurrence and daily schedules of working children in more detail could help clarify this result and unravel other risk factors for injury that working children face [18].

Similar to multiple studies that have demonstrated a positive association between an increased workload and the prevalence of injuries [9,11,16,19,31], our research revealed an increased risk in injury with every additional hour of work per day and with working in more than one job at the same time. Taking breaks during the workday was associated with decreased odds of reporting injuries, as reported elsewhere [59,60]. The results showed modest variations between male and female children. These small differences between males and females may be due to variations in working patterns, including the duration of the breaks, and the different activities and behaviours the children engage in when they are not performing their required tasks [54]. Moreover, even when males and females are performing the same work tasks, different performance expectations may contribute to different exposures and outcomes [37,57]. The occupational health literature has shown that discrepancies in health and safety outcomes between males and females performing the same jobs and tasks may be attributed to different biological, psychological, and environmental factors which incur different responses [29,35]. Another possible factor could be the difference in the duration (exposure-time) and frequency of performing each task for males and females; however, evidence on such measures, particularly in the context of child labor, remains lacking [37].

The use of sharp or heavy objects was also significantly associated with the occurrence of injury among working children. The lack of proper safety training for the use of tools and machinery in agricultural work and other jobs contributes significantly to poor work safety and increased risk of injuries and adverse outcomes among working children [61]. Children facing additional pressure to complete their job on time showed significantly higher odds of injury than those who did not report working under pressure. It has been proven that children are particularly vulnerable to exploitation by employers, who take advantage of their lack of expertise, limited authority, and lower wages to increase production and profit [27,62,63]. Our results concur with those of previous studies that showed an increased risk of injury among children compensated with piece-rate pay [27,62]. Children who have to complete a specific number of tasks/items in a working day, endure longer working hours and harsher conditions to increase their earnings at the expense of their own safety [63]; this may result in increased risk of injury [27,62]. Exploitation of child workers can also manifest in physical abuse and violence, which has been reported in the literature [22]. Almost a quarter of the children in our study reported being physically abused on the job, and they were more than twice as likely to sustain injuries as other children. This reflects the alarming exploitation and harm many children are enduring, which remains under-reported [26].

According to the literature, risks for occupational injury among working children are not limited to those directly caused on the job, but can also occur during the commute to and from the job site [34]. In accordance with a study conducted in Mexico [34], our findings showed that children who reported pickup trucks as the means of transportation to and from work had lower odds of injury occurrence than those who walk or cycle; this may be due to falls and vehicle-pedestrian collisions, particularly in areas with poor infrastructure and lack of road safety [64].

The results of our study raise an alarm: the precarious working conditions of refugee children described in this study are expected to worsen. The socio-economic conditions of Syrian refugees have been greatly impacted by the evolving situation in Lebanon and globally. Since October 17, 2019, Lebanon has witnessed a state of deteriorating unrest and severe economic strife that have further impacted the livelihoods of the population, including that of Syrian refugees [30,65,66]. This was further compounded by the repercussions of the global COVID-19 pandemic, which put the local economy at a standstill with the closure of many businesses and sectors, and forced huge numbers of people into unemployment [67]. With the critical deterioration of the local currency, hyperinflation, and tremendous increase in prices of basic goods, more families are struggling to make ends meet and provide the most basic needs [30,65,66]. This is projected to push an already marginalized population of locals and Syrian refugees further into extreme poverty [67]. Accordingly, more Syrian refugee children will be forced into labor to provide support to their impoverished families at the expense of their education putting them at risk of hazardous working conditions and exploitation [67]. It is expected that both male and female children will be further affected by deteriorating work conditions; consequently, understanding the gender-related differences in risks and exposures is critical to devise the needed interventions and policies and properly curtail negative health outcomes.

In order to mitigate the deprivation of Syrian refugee families, international aid and social initiatives could focus on sustainable goals that provide long-term support. This can be done through training and capacity building initiatives to improve the skills of family breadwinners and expand their opportunities in providing for their families. In addition, public awareness campaigns on safety measures and proper handling of materials, tools, and machinery targeting employers and workers of varying age-groups could ensure safer working environments [62]. The need to create an adequate safety climate is essential to prevent the occurrence of work-place accidents and injury and curtail the impact on the health of working children [29,61,62]. Training efforts would be more effective if carried out on a periodic basis, as one-time initiatives have shown little retention of knowledge after a period of time and consequently low levels of compliance [57,68]. Introducing such initiatives into school curricula and community outreach initiatives could help ensure better reach and impact [32]. This can be coupled with incentives to ensure consistent school attendance [32] by providing monetary assistance to families that send their children to school.

Our study has a number of limitations. It relies on self-reported exposure and outcome measures, and may be prone to recall or social desirability bias, and hesitation to answer sensitive questions about physical abuse and work pressure. However, interviewing the children in their home may have provided some reassurance and willingness to share their work experience. In addition, the selection of children living in tented refugee settlements could have biased the sample towards the most socioeconomically vulnerable portion of the Syrian refugee population in Lebanon, thus limiting generalizability. However, given that it is one of the largest studies to be conducted on this population, it provides substantial knowledge on the conditions endured by refugee child workers in Lebanon. Moreover, the large and relatively even gender balance in our sample enabled an insightful gender-stratified analysis, which is an important contribution to the literature on child labor that often under-sample females [31,49,62]. Finally, in assessing injury ever experienced at work, our results did not account for unequal exposure periods at work and may not reflect the changing working conditions that children could encounter over multiple work experiences. Children who reported multiple injuries did not specify whether these injuries occurred in a single or multiple injury events. This highlights the need for additional observational studies and particularly longitudinal designs which could help reveal the evolving situation of child workers over time and better track injury events as they occur.

## Conclusion

Our findings showed an association between the occurrence of injury and multiple risk factors for Syrian refugee children in Lebanon, highlighting the strenuous working conditions they endure including the prolonged working hours, the use of sharp and heavy materials, and experiencing pressure to complete tasks during a limited amount of time. It also sheds light on the alarming prevalence of physical abuse among working children, which reflects the rampant violation of children’s rights. Many injuries whether caused by the improper use of tools, by accidents, or poor management of equipment can be prevented through direct safety interventions and the proper implementation of child labor policies. It is evident that further research is needed to reduce the gap in documentation on the health and safety of Syrian refugee child workers, which can further inform policies and direct more effective strategies. Research that adopts a gender-sensitive approach would better identify problems particular to males and females which have been generally overlooked and would require customized interventions. Interventions could include organizing work safety trainings for employers, families, and workers to promote better work safety climates in settings where children’s work perpetuates. In addition, providing accessible schooling with incentives for attendance, as well as ensuring sustainable family support through employment opportunities for care-givers and aid to secure basic needs, are critical to ensure effective change initiatives. Multidimensional interventions are essential to address the complex evolving challenges facing refugees and other disadvantaged populations.

## Supporting information

S1 STROBE checklist STROBE, strengthening the reporting of observational studies in epidemiology. (DOCX)

S1 Table Associations between sociodemographic, work characteristics and work-related injuries among male Syrian refugee working children (8–18 years) in the Bekaa Valley, Lebanon, 2017 (N = 2107). (DOCX)

S2 Table Associations between sociodemographic, work characteristics and work-related injuries among female Syrian refugee working children (8–18 years) in the Bekaa Valley, Lebanon, 2017 (N = 1983). (DOCX)

S1 Text Questions and answer choices. (DOCX)
