# Supplementary material for: Work-related injuries among Syrian refugee child workers in the Bekaa Valley of Lebanon: A gender-sensitive analysis
Source: PLoS One. 2021 Sep 20;16(9):e0257330. doi: 10.1371/journal.pone.0257330 (PMC8452013; doi:10.1371/journal.pone.0257330)
Supplement: S1 Table — (DOCX) [file pone.0257330.s002.docx]

**S1Table.**

**Associations between sociodemographic, work characteristics and work-related injuries among male Syrian refugee working children (8 – 18 years) in the Bekaa Valley, Lebanon, 2017 (N= 2107)**

| Injured (753, 35.7 %) | | | |
| --- | --- | --- | --- |
| Independent variable |  | **Unadjusted OR (95% CI) (*p*-value)** | **AOR (95% CI) (*p*-value)*** |
|  | **Mean (SD)** |  |  |
| Number of years since child started working | 2.2 (1.5) | 1.13 (1.07-1.21) (<0.001) | 1.08 (1.01-1.16) (0.035) |
| Average work hours/day | 7.6 (2.7) | 1.14 (1.10-1.18) (<0.001) | 1.08 (0.99-1.17) (0.085) |
|  | **% (N)** |  |  |
| Attending school |  |  |  |
| No | 79.8 (601) | 1 | 1 |
| Yes | 20.2 (152) | 1.04 (0.84-1.31) (0.704) | 1.28 (0.97-1.70) (0.084) |
| Transportation to work† |  |  |  |
| Walking | 37.7 (283) | 1 | 1 |
| Cycling | 3.5 (26) | 1.67 (0.94-2.96) (0.081) | 1.47 (0.82-2.65) (0.197) |
| Pickup truck | 58.9 (442) | 0.77 (0.64-0.93) (0.006) | 0.65 (0.51-0.83) (0.001) |
| Working in more than one job† |  |  |  |
| No | 89.4 (672) | 1 | 1 |
| Yes | 10.6 (80) | 2.18 (1.56-3.05) (<0.001) | 1.62 (1.07-2.46) (0.023) |
| Piece-rate pay |  |  |  |
| No | 82.3 (620) | 1 | 1 |
| Yes | 17.7 (133) | 1.16 (0.92-1.47) (0.216) | 1.31 (0.99-1.74) (0.058) |
| Working under pressure to finish job on time |  |  |  |
| No | 36.3 (273) | 1 | 1 |
| Yes | 63.8 (480) | 1.86 (1.55-2.23) (<0.001) | 1.51 (1.19-1.92) (0.001) |
| Taking breaks during workday |  |  |  |
| No | 17.9 (135) | 1 | 1 |
| Yes | 82.1 (618) | 0.88 (0.70-1.10) (0.261) | 0.95 (0.70-1.28) (0.736) |
| Use of sharp/heavy objects at work |  |  |  |
| No | 53.7 (404) | 1 | 1 |
| Yes | 46.4 (349) | 2.40 (1.99-2.90) (<0.001) | 1.89 (1.49-2.39) (<0.001) |
| Physically abused at work |  |  |  |
| No | 72.1 (543) | 1 | 1 |
| Yes | 27.9 (210) | 2.64 (2.11-3.31) (<0.001) | 2.95 (2.23-3.89) (<0.001) |

*Model clustered at the household level and adjusted for age.
†Due to respondents answering ‘I don’t know’ or respondents not answering, the total does not add up to 753.

Abbreviations: OR: odds ratio; AOR, adjusted odds ratio; CI, confidence interval; SD: standard deviation
