# Supplementary material for: Work-related injuries among Syrian refugee child workers in the Bekaa Valley of Lebanon: A gender-sensitive analysis
Source: PLoS One. 2021 Sep 20;16(9):e0257330. doi: 10.1371/journal.pone.0257330 (PMC8452013; doi:10.1371/journal.pone.0257330)
Supplement: S2 Table — (DOCX) [file pone.0257330.s003.docx]

**S2Table.**

**Associations between sociodemographic, work characteristics and work-related injuries among female Syrian refugee working children (8 – 18 years) in the Bekaa Valley, Lebanon, 2017 (N= 1983)**

| Injured (530, 26.7%) | | | |
| --- | --- | --- | --- |
| Independent variable |  | **Unadjusted OR (95% CI) (*p*-value)** | **AOR (95% CI) (*p*-value)*** |
|  | **Mean (SD)** |  |  |
| Number of years since child started working | 2.3 (1.6) | 1.20 (1.12-1.28) (<0.001) | 1.19 (1.10-1.29) (<0.001) |
| Average work hours/day | 7.0 (2.8) | 1.12 (1.07-1.16) (<0.001) | 1.07 (1.02-1.12) (0.008) |
|  | **% (N)** |  |  |
| Attending school |  |  |  |
| No | 83.0 (440) | 1 | 1 |
| Yes | 17.0 (90) | 1.04 (0.80-1.36) (0.750) | 1.33 (0.94-1.89) (0.103) |
| Transportation to work† |  |  |  |
| Walking | 19.0 (98) | 1 | 1 |
| Cycling | 0.4 (2) | 1.61 (0.29-8.94) (0.585) | 2.30 (0.41-12.99) (0.345) |
| Pickup truck | 80.6 (416) | 1.22 (0.95-1.57) (0.585) | 0.79 (0.56-1.10) (0.165) |
| Working in more than one job |  |  |  |
| No | 95.9 (508) | 1 | 1 |
| Yes | 4.2 (22) | 2.05 (1.17-3.59) (0.012) | 1.87 (0.98-3.56) (0.056) |
| Piece-rate pay |  |  |  |
| No | 86.2 (457) | 1 | 1 |
| Yes | 13.8 (73) | 1.02 (0.77-1.37) (0.870) | 1.12 (0.79-1.60) (0.524) |
| Working under pressure to finish job on time |  |  |  |
| No | 27.9 (148) | 1 | 1 |
| Yes | 72.1 (382) | 2.38 (1.92-2.95) (<0.001) | 1.78 (1.35-2.35) (<0.001) |
| Taking breaks during workday |  |  |  |
| No | 15.7 (83) | 1 | 1 |
| Yes | 84.3 (447) | 0.82 (0.62-1.06) (0.142) | 0.92 (0.67-1.25) (0.578) |
| Use of sharp/heavy objects at work |  |  |  |
| No | 49.8 (264) | 1 | 1 |
| Yes | 50.2 (266) | 2.13 (1.74-2.61) (<0.001) | 1.86 (1.46-2.36) (<0.001) |
| Physically abused at work |  |  |  |
| No | 82.8 (439) | 1 | 1 |
| Yes | 17.2 (91) | 2.22 (1.66-2.97) (<0.001) | 1.91 (1.32-2.74) (0.001) |

*Model clustered at the household level and adjusted for age.
†Due to respondents answering ‘I don’t know’ or respondents not answering, the total does not add up to 530.

Abbreviations: OR: odds ratio; AOR, adjusted odds ratio; CI, confidence interval; SD: standard deviation
