# Supplementary material for: Work-related injuries among Syrian refugee child workers in the Bekaa Valley of Lebanon: A gender-sensitive analysis
Source: PLoS One. 2021 Sep 20;16(9):e0257330. doi: 10.1371/journal.pone.0257330 (PMC8452013; doi:10.1371/journal.pone.0257330)
Supplement: S1 Text — (DOCX) [file pone.0257330.s004.docx]

**S1 Text. Questions and Answer Choices**

| **Questions** | **Answer choices** | |
| --- | --- | --- |
| **Sociodemographic characteristics** | | |
| Household ID | Enter an integer | |
| How old are you? [Age] | Enter an integer | |
| Gender | 1. Male  2. Female | |
| **Schooling** |  | |
| Do you currently go to school? | 0. No  1. Yes | |
| **Work Information** |  | |
| How old were you when you started working? | Enter an integer | |
| How long have you been working at your current job? | Enter an integer (years)  98. No answer | |
| How many hours per day do you work? | Enter an integer (hours/day)  98. No answer | |
| Do you currently work in more than one job? | 0. No  1. Yes | |
| What is your field of work? | Multiple options allowed | |
| Agriculture | 0. No  1. Yes | |
| Waste picking | 0. No  1. Yes | |
| Vending and delivery | 0. No  1. Yes | |
| Craft and related trade work | 0. No  1. Yes | |
| Construction | 0. No  1. Yes | |
| Street services | 0. No  1. Yes | |
| Factory work | 0. No  1. Yes | |
| Car wash | 0. No  1. Yes | |
| Mechanics | 0. No  1. Yes | |
| Miscellaneous jobs | 0. No  1. Yes | |
| What tasks do you perform in agriculture? | Multiple options allowed | |
| Harvesting | 0. No  1. Yes | |
| Loading/Carrying | 0. No  1. Yes | |
| Weeding | 0. No  1. Yes | |
| Cultivating | 0. No  1. Yes | |
| Planting | 0. No  1. Yes | |
| Food sorting | 0. No  1. Yes | |
| Livestock keeping | 0. No  1. Yes | |
| How do you go to/come from work (transportation)? | 1. Walking  2. Cycling  3. Pickup truck  96. Other, specify | |
| **Work Injuries** |  | |
| While working, have you ever suffered any injuries due to: (Multiple options allowed) | Answer | Hospitalized injury |
| Falls/slips/trips | 0. No  1. Yes | 0. No  1. Yes |
| Insect/animal bites | 0. No  1. Yes | 0. No  1. Yes |
| Cuts/wounds | 0. No  1. Yes | 0. No  1. Yes |
| Falling objects | 0. No  1. Yes | 0. No  1. Yes |
| Electrocution/burns | 0. No  1. Yes | 0. No  1. Yes |
| Fractures/Sprains | 0. No  1. Yes | 0. No  1. Yes |
| Eye injuries | 0. No  1. Yes | 0. No  1. Yes |
| Other, specify | 0. No  1. Yes | 0. No  1. Yes |
| **Work Conditions** |  | |
| Do you take breaks during work hours? | 0. No  1. Yes | |
| Do you use sharp/heavy objects while working? | 0. No  1. Yes | |
| During work, have you ever been physically abused? | 0. No  1. Yes | |
| Are you paid on a piece-rate basis? | 0. No  1. Yes | |
| Do you work under pressure to finish your job on time? | 0. No  1. Yes | |
